# A Composite Endpoint of Liver Surgery (CELS): Development and Validation of a Clinically Relevant Endpoint Requiring a Smaller Sample Size

**DOI:** 10.1245/s10434-025-16965-y

**Published:** 2025-01-31

**Authors:** Jun Kawashima, Miho Akabane, Yutaka Endo, Selamawit Woldesenbet, Mujtaba Khalil, Kota Sahara, Andrea Ruzzenente, Luca Aldrighetti, Todd W. Bauer, Hugo P. Marques, Rita Lopes, Sara Oliveira, Guillaume Martel, Irinel Popescu, Mathew J. Weiss, Minoru Kitago, George Poultsides, Kazunari Sasaki, Shishir K. Maithel, Tom Hugh, Ana Gleisner, Federico Aucejo, Carlo Pulitano, Feng Shen, François Cauchy, Bas Groot Koerkamp, Itaru Endo, Timothy M. Pawlik

**Affiliations:** 1 Department of Surgery, The Ohio State University Wexner Medical Center and James Comprehensive Cancer Center, Columbus, OH USA; 2 Department of Gastroenterological Surgery, Yokohama City University School of Medicine, Yokohama, Japan; 3 Department of Transplant Surgery, University of Rochester Medical Center, Rochester, NY USA; 4 Department of Surgery, University of Verona, Verona, Italy; 5 Department of Surgery, Ospedale San Raffaele, Milan, Italy; 6 Department of Surgery, University of Virginia, Charlottesville, VA USA; 7 Department of Surgery, Curry Cabral Hospital, Lisbon, Portugal; 8 Department of Surgery, University of Ottawa, Ottawa, ON Canada; 9 Department of Surgery, Fundeni Clinical Institute, Bucharest, Romania; 10 Department of Surgery, Northwell Health, New Hyde Park, NY USA; 11 Department of Surgery, Keio University, Tokyo, Japan; 12 Department of Surgery, Stanford University, Stanford, CA USA; 13 Department of Surgery, Emory University, Atlanta, GA USA; 14 Department of Surgery, The University of Sydney, Sydney, NSW Australia; 15 Department of Surgery, University of Colorado Denver, Denver, CO USA; 16 Department of General Surgery, Cleveland Clinic Foundation, Cleveland, OH USA; 17 Department of Surgery, Royal Prince Alfred Hospital, Camperdown, NSW Australia; 18 Department of Surgery, Eastern Hepatobiliary Surgery Hospital, Shanghai, China; 19 Department of Hepatobiliopancreatic Surgery, APHP, Beaujon Hospital, Clichy, France; 20 Department of Surgery, Erasmus University Medical Centre, Rotterdam, The Netherlands

## Abstract

**Background:**

The feasibility of trials in liver surgery using a single-component clinical endpoint is low because single endpoints require large samples due to their low incidence. The current study sought to develop and validate a novel composite endpoint of liver surgery (CELS) to facilitate the generation of more feasible and robust high-level evidence in the field of liver surgery.

**Methods:**

Patients who underwent curative-intent hepatectomy for hepatocellular carcinoma, intrahepatic cholangiocarcinoma, or colorectal liver metastasis were identified using a multi-institutional database. Components of CELS were selected based on perioperative liver surgery-specific complications using univariable logistic regression models. The association of CELS with prolonged length of stay (LOS) and surgery-related death was evaluated and externally validated. Sample sizes were calculated for both individual outcomes and CELS.

**Results:**

Among 1958 patients, 377 (19.3%) met CELS criteria based on postoperative bile leak (*n* = 221, 11.3%), post-hepatectomy liver failure (*n* = 71, 3.6%), post-hepatectomy hemorrhage (*n* = 38, 1.9%), or intraoperative blood loss of 2000 ml or greater (*n* = 101, 5.2%). CELS demonstrated favorable discriminative accuracy of surgery-related death (analytic cohort: area under the curve [AUC], 0.79 vs external validation cohort: AUC, 0.85). In addition LOS was longer among the patients with a positive CELS (analytic cohort: 14 vs. 9 days [*p* < 0.001] vs. the validation cohort: 10 vs. 6 days [*p* < 0.001]). Relative to individual endpoints, CELS allowed a 45.8–91.6% reduction in sample size.

**Conclusion:**

CELS effectively predicted surgery-related death and can be used as a standardized, clinically relevant endpoint in prospective trials, facilitating smaller sample sizes and enhancing feasibility compared with single quality outcome metrics.

**Supplementary Information:**

The online version contains supplementary material available at 10.1245/s10434-025-16965-y.

Primary liver cancer, which includes hepatocellular carcinoma (HCC) and intrahepatic cholangiocarcinoma (ICC), is the fifth leading cause of cancer-related mortality in the United States.^1,2^ In 2023, primary liver cancer was diagnosed in 41,630 new cases, and 29,840 individuals died of primary liver cancer.^1^ Moreover, about 50% of patients with colorectal cancer, the second leading cause of cancer deaths in the United States, experience colorectal liver metastases (CRLM), which further complicates the management of this disease.^1,3^ Although liver resection remains the cornerstone of curative-intent treatment for primary and secondary liver malignancies, a subset of patients will experience perioperative morbidity.^4^ This morbidity is due to a variety of general complications as well as liver surgery-specific complications, including postoperative bile leak, post-hepatectomy liver failure (PHLF), and post-hepatectomy hemorrhage (PHH).^5–7^ Notably, these complications can adversely affect patient quality of life, leading to longer hospital stays, higher rates of readmission, and compromised long-term survival.^4–7^ Consequently, the ongoing development of safer and oncologically effective surgical techniques for liver surgery is crucial.

Conventional approaches to evaluating the quality of surgical techniques have focused on individual outcome variables such as blood loss, specific morbidity and mortality rates, reoperation, and hospital length of stay (LOS).^8–10^ Although these individual metrics provide valuable insights, they do not fully capture the comprehensive quality of surgical procedures.^11^ In modern liver surgery, the incremental advancements in techniques and the decreasing incidence of individual complications due to improvements in surgical and perioperative care pose additional challenges.^12^ The infrequent occurrence of these discrete outcomes necessitates large sample sizes to detect statistically meaningful differences, which makes conducting robust randomized controlled trials (RCTs) or other prospective studies impractical.^13^ This reliance on isolated endpoints also constrains the ability to evaluate surgical outcomes comprehensively, underscoring the need for alternative methods of assessment that can more accurately reflect the multifaceted nature of surgical success.^11–13^

To address these limitations, several researchers have proposed the use of composite endpoints.^13^ A composite endpoint consists of at least two distinct outcomes. By combining multiple endpoints, the overall event rate can be increased.^13^ In cardiology, the adoption of composite endpoints has led to meaningful studies with adequate statistical power based on clinically relevant metrics.^14,15^ Similarly, establishing a widely applicable, clinically meaningful, and internationally accepted composite endpoint would be highly beneficial in liver surgery. Therefore, the current study sought to develop and validate a novel Composite Endpoint of Liver Surgery (CELS). This initiative is intended to facilitate the generation of more feasible and robust high-level evidence in the field of liver surgery, ultimately improving patient outcomes and advancing surgical practices.

## Methods

### Data Source and Patient Selection

Patients who underwent curative-intent liver resection for HCC, ICC, or CRLM between 2000 and 2023 were identified from an international multi-institutional database.^16–18^ The study excluded individuals who underwent palliative surgery or had missing data on blood loss, complication, LOS, recurrence, or follow-up evaluation. The study was approved by the institutional review board of each participating institution.

### Variables of Interest

Information on patient clinicopathologic characteristics such as age, sex, American Society of Anesthesiology physical status (ASA-PS) classification (i.e., ≤2 or >2), year of surgery (i.e., 2000–2010 or 2011–2023), histology (i.e., HCC, ICC, or CRLM), type of surgical procedure (i.e., wedge resection, segmentectomy/sectionectomy, left hepatectomy, right hepatectomy, extended left hepatectomy, extended right hepatectomy, or central hepatectomy), use of minimally invasive surgery (MIS), surgical margin (i.e., R0/1 or R2), intraoperative blood loss, occurrence of bile leak, PHLF, PHH, any complications, LOS, and surgery-related death was collected. Definitions of the International Study Group of Liver Surgery (ISGLS) were used for liver surgery-specific complications, including bile leak, PHLF, and PHH.^5–7^ The severity of other complications was defined according to the Clavien-Dindo classification system (grades I–V). Severe complications were defined as Clavien-Dindo classification ≥III.^19^

After curative-intent resection, patients were monitored every 3 to 4 months for the first 2 years, then every 6 months thereafter. During follow-up evaluation, patients were monitored by serum tumor markers (AFP, CEA, or CA19-9) and imaging examinations (computed tomography, and/or magnetic resonance imaging).^16–18^ Overall survival (OS) was defined as the time elapsed between the date of liver resection and death. Treatment of tumor recurrence was based on consensus among the multidisciplinary team at each institution.

### Outcome and Predictors

The primary outcome was surgery-related death, defined as death from any complications within 90 days after surgery. The secondary outcome was LOS, defined as the number of days from surgery to hospital discharge. Hospital length of stay was determined as the dependent variable, LOS and dichotomized by dividing the outcome into a prolonged stay, defined as 16 days or longer (above the 75th percentile), and a regular stay, defined as 15 days or shorter. Predictor variables were predefined and based on clinical relevance. To provide an easily and broadly applicable endpoint for clinicians, the major known contributors to postoperative morbidity and mortality, namely, liver surgery-specific complications such as bile leak, PHLF, and PHH, were included. In addition to these three outcomes, blood loss of 2000 ml or more was included because several studies included blood loss or intraoperative complications with outcomes.^20–22^ The cutoff for blood loss of more than 2000 ml was determined with reference to the Oslo classification.^23^ The composite endpoint of liver surgery (CELS) was defined as positive (1) if a patient experienced one or more of the complications (blood loss ≥2000 ml, bile leak, PHLF, PHH) or negative (0) if none of the complications were present.

### Statistical Analysis

Continuous variables are reported as median values with interquartile ranges (IQRs), whereas categorical variables are reported as frequency/percentages. Univariable logistic regression was used to assess the association between the clinically relevant predictor variables and surgery-related death or prolonged LOS. Predictor variables associated with surgery-related death or prolonged LOS were selected as components of CELS. Then, CELS was calculated for each patient in the entire cohort. The model’s ability to discriminate patients with a surgery-related death was evaluated using sensitivity, specificity, and receiver operating characteristic (ROC) curves and the area under the curve (AUC).

The calibration of the CELS to predict surgery-related death was assessed using a logistic regression model. Prolonged stay was compared among patients with positive and negative CELS using the Wilcoxon rank-sum test. Subsequently, the impact of CELS on survival was evaluated using Kaplan-Meier curves and the log-rank test. Additionally, a conditional survival analysis was performed to evaluate the association of CELS with long-term survival outcomes among patients without surgery-related death. The impact of CELS on surgery-related death, prolonged LOS, and OS also was externally validated using data from the University of Verona and Cleveland Clinic (Table S1).

The theoretical sample size of an adequately powered RCT was calculated as the minimum number of patients necessary in each arm of the trial, with an alpha of 0.05 and a beta of 0.20, using two-sided testing. The sample size was calculated assuming a 10%, 20%, 30%, 40%, 50%, or 60% relative reduction in the incidence of either a single component or the a newly developed CELS.

To address potential variability in outcomes across all hospitals (*n* = 18) and further evaluate the robustness of CELS, several additional analyses were performed. First, a linear mixed-effects model was used to account for hospital-level variability, including hospitals as a random effect. The intraclass correlation coefficient was calculated to determine the proportion of total variance in LOS attributable to differences among hospitals.

Furthermore, hospitals were stratified into two groups based on the median LOS, categorized as hospitals with a shorter LOS (*n* = 9) and hospitals with a longer LOS (*n* = 9). Within each group, the Wilcoxon rank-sum test was used to assess differences in LOS between CELS-positive and CELS-negative patients, allowing for evaluation of the consistency of the impact of CELS across hospitals with varying baseline LOS levels.

Additionally, the influence of wedge resection on the discriminatory performance of CELS was assessed by performing subgroup analyses excluding patients who underwent wedge resection. After their exclusion, the AUC, sensitivity, and specificity of CELS for surgery-related death were recalculated for both the analytic cohort and the external validation cohort, providing insights into the robustness of CELS performance in this subgroup.

To further validate the discriminatory power of CELS, an analysis comparing CELS with severe complications defined by the Clavien-Dindo classification (grade III or higher) was performed. The specificity of CELS and severe complications to predict surgery-related mortality within 90 days was compared in both the analytic and external validation cohorts.

All statistical analyses were performed using R version 4.2.3 (R Project for Statistical Computing, Vienna, Austria). All tests were two-sided, and a *p* value lower than 0.05 was considered statistically significant.

## Results

### Patient Characteristics

The analytic cohort included 1958 patients who underwent hepatectomy for HCC (*n* = 563, 28.8%), ICC (*n* = 676, 34.5%), or CRLM (*n* = 719, 36.7%). Median patient age was 65 years (IQR, 56–72 years), and the majority of the patients were male (*n* = 1237, 63.2%). Among the 1958 patients, 809 (41.3%) were classified as ASA class >2. The surgical procedures included wedge resection (*n* = 364, 18.6%), segmentectomy/sectionectomy (*n* = 552, 28.2%), right hepatectomy (*n* = 360, 18.4%), left hepatectomy (*n* = 249, 12.7%), extended right hepatectomy (*n* = 164, 8.4%), extended left hepatectomy (*n* = 111, 5.7%), and central hepatectomy (*n* = 31, 1.6%). Minimally invasive surgery (MIS) was performed in 324 (16.5%) cases. The median intraoperative blood loss was 300 ml (IQR, 100–700 ml), with 101 (5.2%) patients experiencing blood loss exceeding 2000 ml. For a small number of patients (*n* = 16, 0.8%), the resection margin was R2. Approximately one-half of patients (*n* = 923, 47.1%) experienced complications, among whom 375 (19.2%) patients were classified as Clavien-Dindo III, IV, or V. Bile leaks occurred in 221 (11.3%) patients, PHLF in 71 (3.6%) patients, and PHH in 38 (1.9%) patients.

The median hospital stay was 10 days (IQR, 6–15 days), with 458 (23.4%) patients having prolonged LOS (>15 days). A total of 50 (2.6%) patients experienced surgery-related death (Table 1).

**Table 1:** Clinicopathologic characteristics of the analytic cohort

Characteristics	All patients
	(*n* = 1958)
	*n* (%)
Median age: years (IQR)	65 (56–72)
Male sex	1237 (63.2)
ASA class >2	809 (41.3)
Year of surgery, 2011–2023	1561 (79.7)
Histology	
Hepatocellular carcinoma	563 (28.8)
Intrahepatic cholangiocarcinoma	676 (34.5)
Colorectal liver metastasis	719 (36.7)
Surgical procedure	
Wedge resection	364 (18.6)
Segmentectomy/sectionectomy	552 (28.2)
Right hepatectomy	360 (18.4)
Left hepatectomy	249 (12.7)
Extended right hepatectomy	164 (8.4)
Extended left hepatectomy	111 (5.7)
Central hepatectomy	31 (1.6)
Missing	127 (6.5)
Minimally invasive surgery	324 (16.5)
Blood loss: ml (IQR)	300 (100–700)
≥2000 ml	101 (5.2)
Surgical margin, R2	16 (0.8)
Bile leak	221 (11.3)
Grade A	79 (4.0)
Grade B	132 (6.7)
Grade C	10 (0.5)
PHLF	71 (3.6)
Grade A	15 (0.8)
Grade B	23 (1.2)
Grade C	33 (1.7)
PHH	38 (1.9)
Grade A	6 (0.3)
Grade B	16 (0.8)
Grade C	16 (0.8)
CELS	377 (19.3)
Complication	923 (47.1)
Severe complication	375 (19.2)
Hospital stays: days (IQR)	10 (6–15)
>15 days	458 (23.4)
Surgery-related death	50 (2.6)

*IQR*,interquartile range; *ASA* American Society of Anesthesiologists; *PHLF* post-hepatectomy liver failure; *PHH* post-hepatectomy hemorrhage; *CELS* composite endpoint of liver surgery

Data are presented as median (IQR) for continuous measures and *n* (%) for categorical measures

### Development for CELS

Univariable logistic regression models identified factors associated with prolonged hospital stay. Notably, blood loss of 2000 ml or more (odds ratio [OR] 2.36; 95% confidence interval [CI] 1.55–3.55; *p* < 0.001), bile leak (OR 4.04; 95% CI 3.03–5.40; *p* < 0.001), PHLF (OR 2.21; 95% CI 1.34–3.57; *p* = 0.001), and PHH (OR 2.71; 95% CI 1.40–5.18; *p* = 0.003) each were associated with prolonged hospital stays. Several factors also were associated with surgery-related mortality including blood loss of 2000 ml or more (OR 13.65; 95% CI 7.30–25.00; *p* < 0.001), PHLF (OR 36.55; 95% CI 19.53–68.60; *p* < 0.001), and PHH (OR 6.31; 95% CI 2.09–15.62; *p* < 0.001). However, bile leak was not associated with mortality (OR 0.87; 95% CI 0.30–2.02; *p* = 0.771; Table 2).

**Table 2:** Univariable logistic regression analysis of demographic factors associated with hospital length of stay and surgery-related death

Variables	Hospital length of stay		Surgical-related death	
	OR 95% CI	*P* Value^a^	OR 95% CI	*P* Value^a^
Blood loss ≥2000 ml	2.36 (1.55–3.55)	**<0.001**	13.65 (7.30–25.00)	**<0.001**
Bile leak	4.04 (3.03–5.40)	**<0.001**	0.87 (0.30–2.02)	0.771
PHLF	2.21 (1.34–3.57)	**0.001**	36.55 (19.52–68.60)	**<0.001**
PHH	2.71 (1.40–5.18)	**0.003**	6.31 (2.09–15.62)	**<0.001**

*OR* odds ratio; *CI* confidence interval; *PHLF* post-hepatectomy liver failure; *PHH* post-hepatectomy hemorrhage

^a^*P* values in bold font are statistically significant (*p* < 0.05)

All initial parameters (blood loss ≥2,000 ml, bile leak, PHLF, and PHH) were included in CELS due to their association with either prolonged LOS or surgery-related mortality. In addition to liver surgery-specific complications and blood loss, several other factors, including year of surgery, ICC histology, major hepatectomy, MIS, and surgical margin were associated with either prolonged LOS or surgery-related mortality (Table S2). However, because these factors primarily reflected clinicopathologic background, surgical procedure, or oncologic outcomes rather than direct measures of liver surgery-specific perioperative morbidity, they were not included in CELS.

**Fig. 1:**
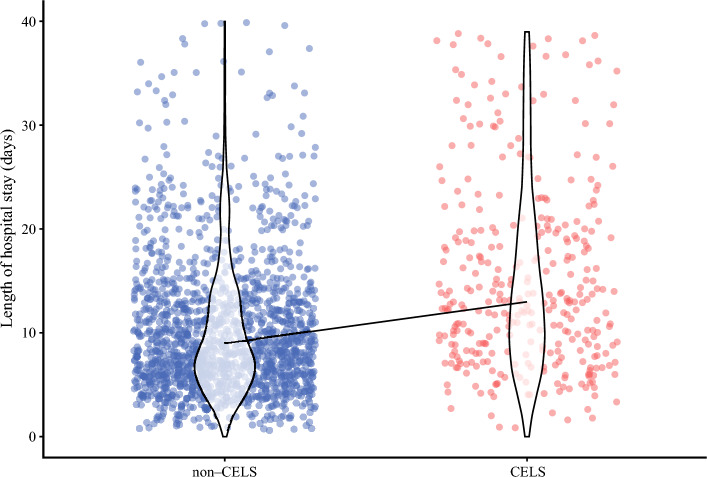
Jitter plot comparing the hospital length of stay between the patients with and without the composite endpoint of liver surgery (CELS) in the analytic cohort

Among the analytic data set, 377 (19.3%) patients were classified as CELS-positive (Table 1). Median LOS differed between the CELS-positive patients (14 days; IQR, 9–24 days) and the CELS-negative patients (9 days; IQR, 6–14 days) (*p* < 0.001; Fig. 1). Notably, CELS demonstrated an AUC of 0.79 (95% CI 0.73–0.85) with a sensitivity of 0.76 and a specificity of 0.82 to predict surgery-related mortality. The calibration curve demonstrated strong agreement between the predicted probability of surgery-related death based on CELS and the observed frequency of surgery-related death in the analytic cohort (Fig. S1A).

### Validation for CELS

In the external validation cohort of 487 patients, 87 (17.9%) were CELS-positive (Table S1). Median LOS was longer for the CELS-positive patients (10 days; IQR, 6–19 days) than for the CELS-negative patients (6 days; IQR, 5–8 days) (*p* < 0.001; Fig. S2). CELS demonstrated a favorable discriminatory ability to predict surgery-related mortality in the external cohort with an AUC of 0.85 (95% CI 0.73–0.98), a sensitivity of 0.88, and a specificity of 0.83. The calibration curve also demonstrated overall good agreement in the external validation cohort (Fig. S1B).

### Assessment of the Impact on Long-Term Outcome

**Fig. 2:**
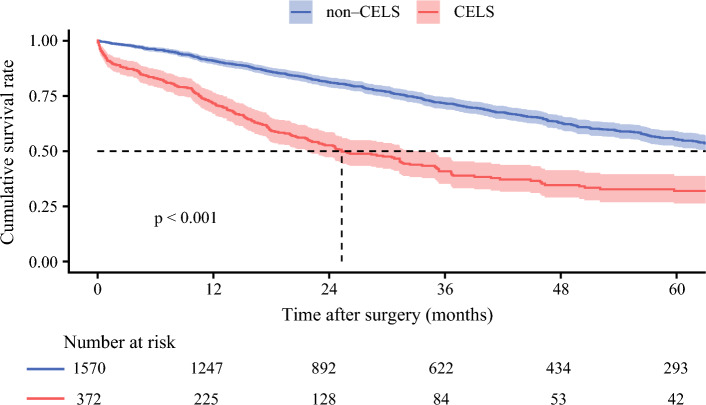
Kaplan-Meier curves demonstrating differences in overall survival between the patients with and without the composite endpoint of liver surgery (CELS) in the analytic cohort

Among 1942 patients with R0 or R1 margins, the median follow-up duration was 25.5 months (IQR, 12.4–48.0 months). The patients with positive CELS had a worse median OS of 25.3 months (95% CI 21.8–33.6 months) compared with 73.3 months (95% CI 65.3–81.0 months) among patients without CELS. There also was a difference in 5-year OS (CELS-positive patients [31.9%] vs CELS-negative patients [55.4%]; *p* < 0.001; Fig. 2). In the external validation cohort of 450 patients without R2 margins, the CELS-positive patients also had worse median OS (49.7 months; 95% CI 23.9–not reached) than CELS-negative patients (52.0 months; 95% CI 44.8–73.0 months; *p* = 0.045; Fig. S3).

After exclusion of the patients with surgery-related death, a conditional survival analysis was performed. In the analytic cohort (*n* = 1892), patients with positive CELS had a worse median OS of 31.3 months (95% CI 25.0–36.8 months) versus 73.7 months (95% CI 66.2–82.2 months) among CELS-negative patients (*p* < 0.001; Fig. S4A). In the external validation cohort (*n* = 444), although no significant difference was observed between CELS-positive patients (median OS, 49.7 months; 95% CI 24.5–not-reached) and CELS-negative patients (median OS, 53.8 months; 95% CI 45.5–73.0 months) (*p* = 0.300), CELS-positive patients exhibited a trend toward worse survival outcomes (Fig. S4B).

### Assessment of the Impact on Sample Size

**Fig. 3:**
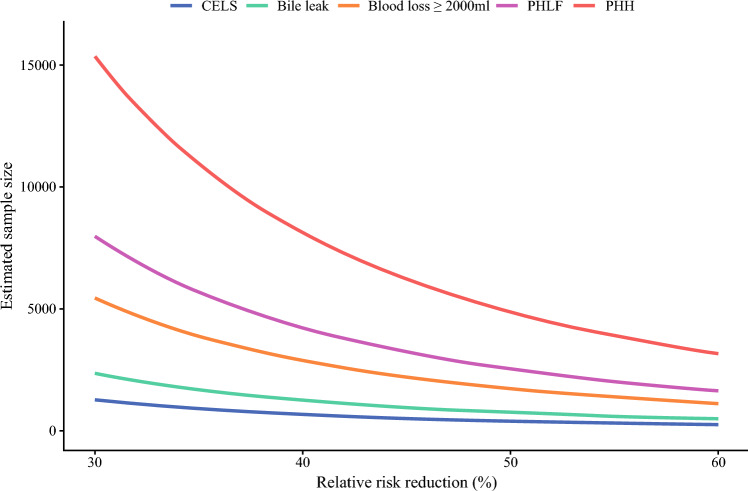
Required sample size for sample size calculation based on the assumed relative risk reduction for each primary outcome parameter

Sample size requirements for clinical trials were calculated based on complication incidence rates to achieve different levels of minimal clinically important risk reductions. Assuming a 10% relative risk reduction, using CELS as the primary endpoint would require a total sample size of 12,613 versus 54,516 using blood loss, 23,554 using bile leak, 80,008 using PHLF, and 154,137 using PHH alone as endpoints. With a 50% relative risk reduction, the required sample sizes were 410 using CELS versus 1733 using blood loss, 756 using bile leak, 2538 using PHLF, and 4879 using PHH alone. These data corresponded to a reduction in sample size of 76.3%, 45.8%, 83.8%, and 91.6% for CELS relative to each individual endpoint, respectively (Table 3) (Fig. 3).

**Table 3:** Required sample size for sample size calculation based on the assumed relative risk reduction for each primary outcome parameter

Relative risk reduction	10%	20%	30%	40%	50%	60%
Blood loss ≥2000 ml	54,516	12,941	5443	2886	1733	1124
Bile leak	23,554	5608	2365	1256	756	490
PHLF	80,008	18,979	7977	4228	2538	1646
PHH	154,137	36,536	16,347	8129	4879	3163
CELS	12,613	3017	1277	680	410	266

*PHLF* post-hepatectomy liver failure; *PHH* post-hepatectomy hemorrhage; *CELS* composite endpoint of liver surgery

### Sensitivity Analyses: Impact of Hospital-Level Variation in LOS and Extent of Hepatic Resection

The linear mixed-effects model demonstrated that hospital-level variation accounted for 18.4% of the total variance in LOS (intraclass correlation coefficient, 0.184), indicating a moderate degree of variation among hospitals. In the sensitivity analysis, hospitals (*n* = 18) were stratified into two groups based on the median LOS. Among hospitals with a shorter LOS (*n* = 9), median LOS was longer among CELS-positive patients (10 days; IQR, 6–19 days) than among CELS-negative patients (6 days; IQR, 4–8 days) (*p* < 0.001). Similarly, among hospitals with a longer LOS (*n* = 9), CELS-positive patients also experienced a longer LOS (19 days; IQR, 12–31.5 days) than CELS-negative patients (13 days; IQR, 9–17 days) (*p* < 0.001). These findings demonstrated that, despite inter-hospital variability in LOS, CELS positivity consistently contributed to a longer LOS in both hospital groups.

To evaluate the influence of wedge resections on the performance of CELS, an additional analysis excluding patients who underwent wedge resection was performed. In the analytic cohort (*n* = 1594), the AUC to predict surgery-related death was 0.79 (95% CI 0.73–0.85) with a sensitivity of 0.79 and a specificity of 0.78. Similarly, in the external validation cohort (*n* = 352), the AUC was 0.83 (95% CI 0.71–0.96) with a sensitivity of 0.88 and a specificity of 0.79.

To further validate the discriminatory ability of CELS, a comparative analysis with severe complications (defined as Clavien-Dindo grade III or higher) was performed. In the analytic cohort, the AUC to predict surgery-related mortality was comparable between CELS and severe complications (0.82 and 0.83, respectively). Similarly, in the external validation cohort, the AUC of CELS was equivalent to that of severe complications (0.83 and 0.84, respectively). These findings underscored that CELS achieved comparable discriminatory performance while maintaining a specific focus on liver surgery-related complications.

## Discussion

Despite advancements in surgical techniques, liver resection for malignancies continues to carry a substantial postoperative morbidity risk, with the incidence of morbidity reported to range from 20 to 45%.^24–28^ These complications can lead to failure-to-rescue, increased in-hospital mortality, non-home discharges, escalated health care costs, and worse long-term survival.^29^ In light of these risks, ongoing efforts to refine surgical techniques that enhance both patient safety and oncologic effectiveness remain essential.

To support the safe introduction of new surgical techniques, establishing reliable methods to assess surgical quality is critical. For liver resection, direct evaluation of techniques through intraoperative and liver-specific complications is theoretically logical.^12^ Although conventional individual endpoints provide important data, they often fail to assess surgical technique comprehensively and typically have low incidence rates, making large sample sizes necessary and limiting their practicality.^12,13^ Overcoming these limitations requires composite endpoints that consolidate key complications to enhance both assessment comprehensiveness and statistical power.^12,13^ As such, the current study was important because we proposed CELS, a novel composite endpoint combining clinically relevant and liver resection-specific complications such as blood loss of 2000 mL or more, bile leak, PHLF, and PHH. Derived from a large international, multi-institutional database and supported by external validation, CELS demonstrated reliability in predicting prolonged LOS and perioperative mortality. Furthermore, CELS allowed for a substantial reduction in the required sample size compared with individual endpoints for clinical trial planning, underscoring its potential for high clinical and scientific relevance.

The proposed CELS metric offers several advantages over conventional individual parameters for evaluating surgical outcomes. First, it enables a comprehensive assessment of perioperative outcomes, providing a more accurate evaluation of liver surgery performance.^13^ Specifically designed to evaluate liver surgery techniques, CELS incorporates multiple relevant parameters unique to liver procedures.^12^ By focusing on these specific aspects, CELS captures a broader range of potential surgical outcomes, ensuring that critical nuances associated with liver surgery are not overlooked. This comprehensive approach may enhance the evaluation of surgical performance, distinguishing it from conventional assessments using each individual endpoint.^11–13^

Second, using a composite endpoint such as CELS can improve statistical power and reduce sample size requirements in clinical trials. This reduction leads to decreased costs and shorter timelines for research studies, making it feasible to conduct robust trials while still achieving clinically relevant results.^12,13^ By concentrating on meaningful patient outcomes, CELS may enable researchers to draw more reliable conclusions from smaller cohorts, ultimately accelerating the translation of findings into clinical practice.^12,13^

Finally, CELS enhances the comparability of outcomes across studies, aligning with the concept of textbook outcome (TO) or benchmark in liver surgery.^13^ Whereas TO or benchmark provides a broad evaluation of surgical quality and highlights interhospital variations, CELS serves as a more focused composite endpoint specifically tailored to outcomes directly related to surgical techniques.^11,30^ This targeted focus allows CELS to maintain cross-study comparability while remaining more relevant to surgical performance. However, the complexity of TO and benchmark measurements, although effective in identifying areas for improvement, limits their practicality as primary endpoints in prospective studies.^13^ Additionally, those metrics can vary by region and population, potentially challenging consistency across studies.^13^ In contrast, CELS consists of four widely accepted, straightforward endpoints, making it a more practical and universally applicable tool for evaluating liver surgery techniques.

In this study, CELS was composed of four key outcomes (blood loss, bile leak, liver failure, and postoperative bleeding), all associated with either surgery-related mortality or longer hospital stay. Intraoperative blood loss is widely recognized as a critical quality indicator and outcome metric in liver surgery because it is an independent factor contributing to increased postoperative morbidity and mortality.^31–33^ Effective control of bleeding is essential for optimal outcomes. Consistent with previous research, the current study noted that blood loss exceeding 2000 ml was linked to both prolonged LOS and surgery-related mortality.^34^

Bile leakage also emerged as an important factor associated with extended LOS. Although perioperative complications and mortality in liver surgery have declined, the incidence of bile leakage has remained relatively high, ranging from 3.1 to 28.0%.^35,36^ Previous data demonstrated that bile leakage can cause severe complications such as postoperative abdominal infection and sepsis, prolonged LOS, and increase treatment costs.^37^ Notably, one meta-analysis identified risk factors for bile leakage, including surgical procedure type, intraoperative blood transfusions, and bleeding, further underscoring the importance of including bile leakage as a key outcome for surgical tecniques.^36^

As another relatively rare but serious complication, PHLF is associated with high mortality.^38^ A large study demonstrated that PHLF criteria were met for 70% of patients who died after liver resection, with PHLF contributing directly to more than half of these deaths.^39^ Causes of PHLF include surgical techniques such as prolonged inflow occlusion, intraoperative blood loss, and extended operative times, as well as patient factors such as future liver remnant volume and underlying liver conditions.^38^ Given these adverse outcomes and known surgery-related risk factors, it may be appropriate to include PHLF as a critical endpoint for evaluating surgical techniques. Similarly, PHH, although infrequent, with a reported incidence of 1% to 8%, remains a significant source of postoperative morbidity.^7^ Frequently, PHH necessitates transfusions or surgical intervention and can result in fatal outcomes in severe cases.^7^ In the current study, PHH was indeed associated with surgery-related mortality, reinforcing its inclusion in CELS.

Thus, these four outcomes (blood loss, bile leak, PHLF, and PHH) were selected for CELS due to their direct relevance to surgical quality and their impact on patient outcomes.

Although CELS is primarily designed to evaluate short-term outcomes, it is noteworthy that differences in long-term outcomes were observed between the patients with positive and negative CELS. This finding aligns with previous studies indicating that complications or achievement of a TO in liver surgery can influence long-term prognosis.^40–43^ For example, Tsilimigras et al.^41^ demonstrated that achieving a TO was independently associated with 26% and 37% reductions in the hazard of death among patients with intrahepatic ICC and HCC, respectively. The explanation of this relationship between short-term postoperative outcomes and long-term prognosis is likely multifactorial, with one key factor being the immune response to intra- and postoperative complications.^43^ Surgical complications can lead to an immunosuppressed state, potentially promoting the growth of residual tumors.^43^ Consequently, minimizing complications in the postoperative period may enhance short-term recovery while also supporting improved long-term cancer-specific outcomes by reducing immunosuppression and the risk of cancer progression.^43^ Therefore, the observed differences in long-term results based on CELS status suggest a close connection between surgical quality, postoperative complications, and both short- and long-term prognoses. These data imply that CELS may serve not only as an indicator of short-term outcomes but also as a valuable predictor of long-term survival for patients undergoing liver surgery.

Several limitations should be considered when the results of this study are interpreted. The retrospective nature of the analysis may have introduced selection bias, potentially influencing treatment decisions. Although the use of an international multi-institutional database offered valuable insights, one notable limitation was the variability in surgical techniques, procedural decisions, and management of complications, which can differ significantly across institutions and regions. The condition of the background liver, including cirrhosis, albumin-bilirubin score, and Child-Pugh classification, is important for perioperative outcomes in liver surgery. However, these data were not available in the current study and therefore were not be included in the analysis. In the conditional survival analysis, no statistically significant difference was observed in the external validation cohort. However, the survival curves consistently demonstrated worse outcomes for the CELS-positive patients than for the CELS-negative patients (Fig. S4B). This finding suggested the possibility of a type 2 error due to the limited sample size of the external validation cohort. Future studies with larger cohorts are needed to further validate these findings and confirm the generalizability of the CELS framework for external populations. Furthermore, the method for quantifying blood loss has not been standardized among the participating centers, potentially leading to variability in the reported outcomes. These factors underscore the importance of exercising caution when the results of this study are interpreted and applied across various clinical settings.

In conclusion, CELS was developed and validated as a valuable tool for assessing outcomes after liver surgery, demonstrating favorable accuracy in predicting perioperative mortality. The clinical relevance of CELS was substantial because it performed well on external validation and may enhance the feasibility of clinical trials by reducing required sample sizes. Establishing a reliable and internationally recognized endpoint for liver surgery has the potential to improve patient outcomes and guide clinical decision-making. In achieving these objectives, CELS may play a crucial role.

## Supplementary Information

Below is the link to the electronic supplementary material.

Supplementary file1 (DOCX 45 KB)

Supplementary file2 (TIF 397 KB)

Supplementary file3 (TIF 323 KB)

Supplementary file4 (TIF 318 KB)

Supplementary file5 (TIF 671 KB)
