# Supplementary material for: A Composite Endpoint of Liver Surgery (CELS): Development and Validation of a Clinically Relevant Endpoint Requiring a Smaller Sample Size
Source: Ann Surg Oncol. 2025 Jan 31;32(5):3505–15. doi: 10.1245/s10434-025-16965-y (PMC11976826; doi:10.1245/s10434-025-16965-y)
Supplement: Supplementary file 1 — Supplementary file1 (DOCX 45 KB) [file 10434_2025_16965_MOESM1_ESM.docx]

**Supplementary Table 1**. Clinicopathological characteristics of the external validation cohort.

| Characteristics | All patients |
| --- | --- |
|  | n=487 |
| Age, years | 66 [59, 73] |
| Sex, male | 342 (70.2) |
| ASA class > 2 | 300 (61.6) |
| Year of surgery, 2011-2023 | 446 (91.6) |
| Histology |  |
| Hepatocellular Carcinoma | 314 (64.5) |
| Intrahepatic cholangiocarcinoma | 88 (18.1) |
| Colorectal liver metastasis | 85 (17.5) |
| Surgical procedure |  |
| Non-anatomical resection | 135 (27.7) |
| Segmentectomy | 185 (37.9) |
| Right hepatectomy | 77 (15.8) |
| Left hepatectomy | 37 (7.6) |
| Extended right hepatectomy | 25 (5.1) |
| Extended left hepatectomy | 20 (4.1) |
| Central hepatectomy | 5 (1.0) |
| Missing | 3 (0.6) |
| Minimally invasive surgery | 227 (46.6) |
| Blood loss, ml | 300 [150, 600] |
| > 2000 ml | 28 (5.7) |
| Surgical margin, R2 | 37 (7.6) |
| Bile leak | 38 (7.8) |
| Grade A | 12 (2.5) |
| Grade B | 20 (4.1) |
| Grade C | 6 (1.2) |
| PHLF | 32 (6.6) |
| Grade A | 9 (1.8) |
| Grade B | 18 (3.7) |
| Grade C | 5 (1.0) |
| PHH | 8 (1.6) |
| Grade A | 1 (0.2) |
| Grade B | 3 (0.6) |
| Grade C | 4 (0.8) |
| CELS | 87 (17.9) |
| Complication | 246 (50.5) |
| Severe complication | 375 (19.2) |
| Length of hospital stays, days | 6 [5, 9] |
| Surgery-related death | 8 (1.6) |

Data are presented as median (IQR) for continuous measures and n (%) for categorical measures.

Abbreviations: ASA, American society of Anesthesiologists; PHLF, post hepatectomy liver failure; PHH, post hepatectomy hemorrhage; CELS, composite endpoint of liver surgery

**Supplementary Table 2**. Univariable logistic regression analysis of demographic factors, excluding liver surgery-specific complications and intraoperative blood loss, associated with hospital length of stay and surgery-related death.

|  | length of hospital stays | |  | Surgical-related death | |
| --- | --- | --- | --- | --- | --- |
| Variables | OR 95%CI | *P* value |  | OR 95%CI | *P* value |
| Age | 1.00 [0.99, 1.01] | 0.920 |  | 0.99 [0.97, 1.02] | 0.477 |
| Sex, male |  |  |  |  |  |
| Female | Ref |  |  | Ref |  |
| Male | 0.95 [0.76, 1.18] | 0.630 |  | 0.62 [0.35, 1.10] | 0.100 |
| ASA classification > 2 |  |  |  |  |  |
| ≤ 2 | Ref |  |  | Ref |  |
| > 2 | 1.10 [0.83, 1.46] | 0.515 |  | 1.50 [0.84, 2.68] | 0.170 |
| Year of surgery |  |  |  |  |  |
| 2000-2010 | Ref |  |  | Ref |  |
| 2011-2023 | 0.93 [0.72, 1.21] | 0.583 |  | 0.37 [0.21, 0.67] | **0.001** |
| Histology |  |  |  |  |  |
| Hepatocellular Carcinoma | Ref |  |  | Ref |  |
| Intrahepatic cholangiocarcinoma | 3.87 [2.92, 5.18] | **<0.001** |  | 4.23 [2.33, 8.34] | **<0.001** |
| Colorectal liver metastasis | 1.32 [0.97, 1.80] | 0.082 |  | 0.58 [0.24, 1.38] | 0.223 |
| Surgical procedure |  |  |  |  |  |
| Minor hepatectomy | Ref |  |  | Ref |  |
| Major hepatectomy | 1.56 [1.23, 1.98] | **<0.001** |  | 3.65 [1.67, 9.61] | **0.003** |
| MIS vs. Open |  |  |  |  |  |
| Open surgery | Ref |  |  | Ref |  |
| MIS | 0.32 [0.22, 0.46] | **<0.001** |  | 0.06 [0.00, 0.28] | **0.006** |
| Margin |  |  |  |  |  |
| R0 | Ref |  |  | Ref |  |
| R1/2 | 1.56 [1.16, 2.09] | **0.003** |  | 2.04 [0.98, 3.91] | **0.041** |

P-values in bold font are statistically significant (p < 0.05).

Abbreviations: ASA, American Society of Anesthesiologists; MIS, minimally invasive surgery
