# Supplementary figures and images for: A Composite Endpoint of Liver Surgery (CELS): Development and Validation of a Clinically Relevant Endpoint Requiring a Smaller Sample Size
Source: Ann Surg Oncol. 2025 Jan 31;32(5):3505–15. doi: 10.1245/s10434-025-16965-y (PMC11976826; doi:10.1245/s10434-025-16965-y)

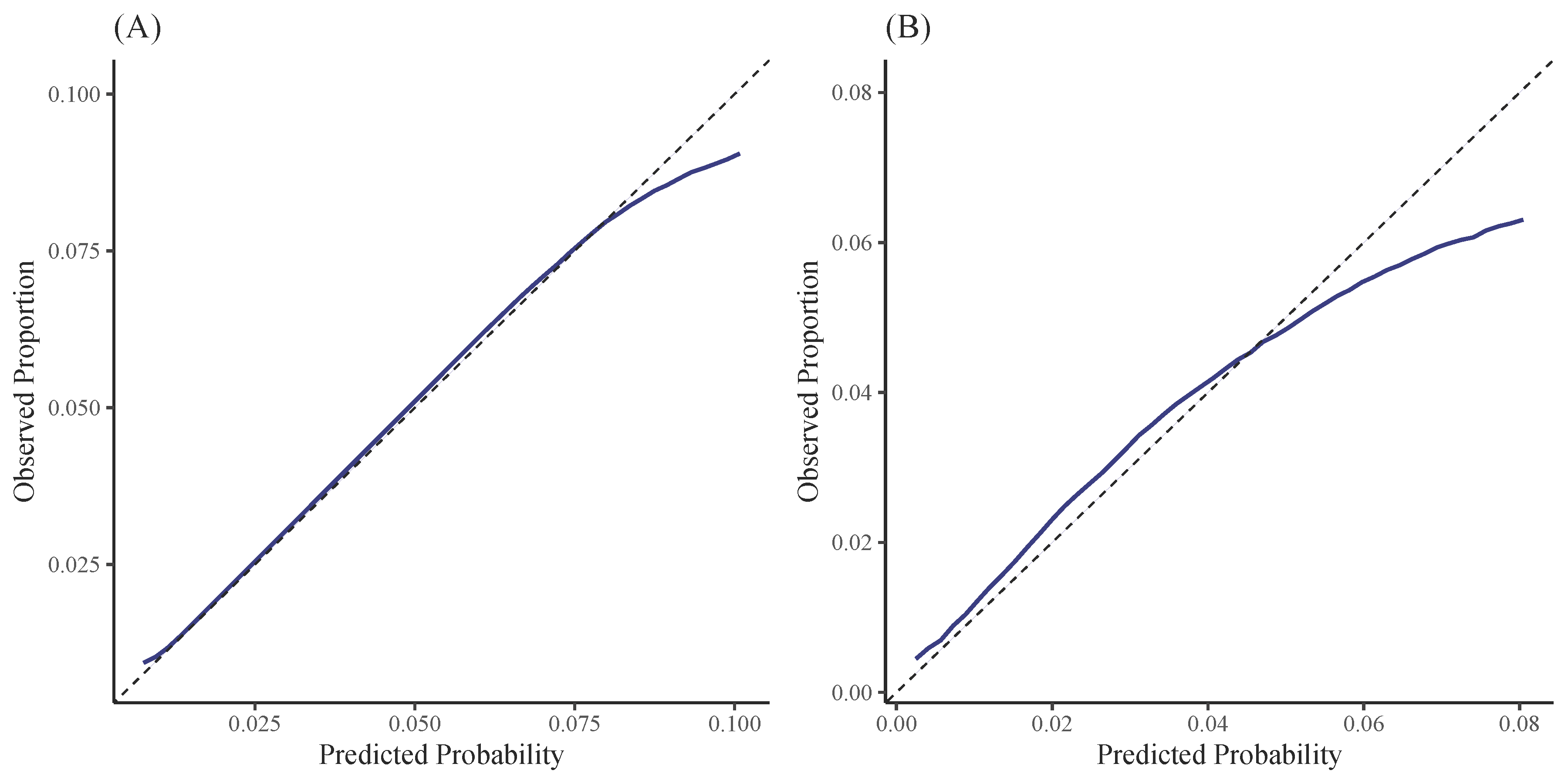

Supplement: Supplementary file 2 — Supplementary file2 (TIF 397 KB) [file 10434_2025_16965_MOESM2_ESM.tif]

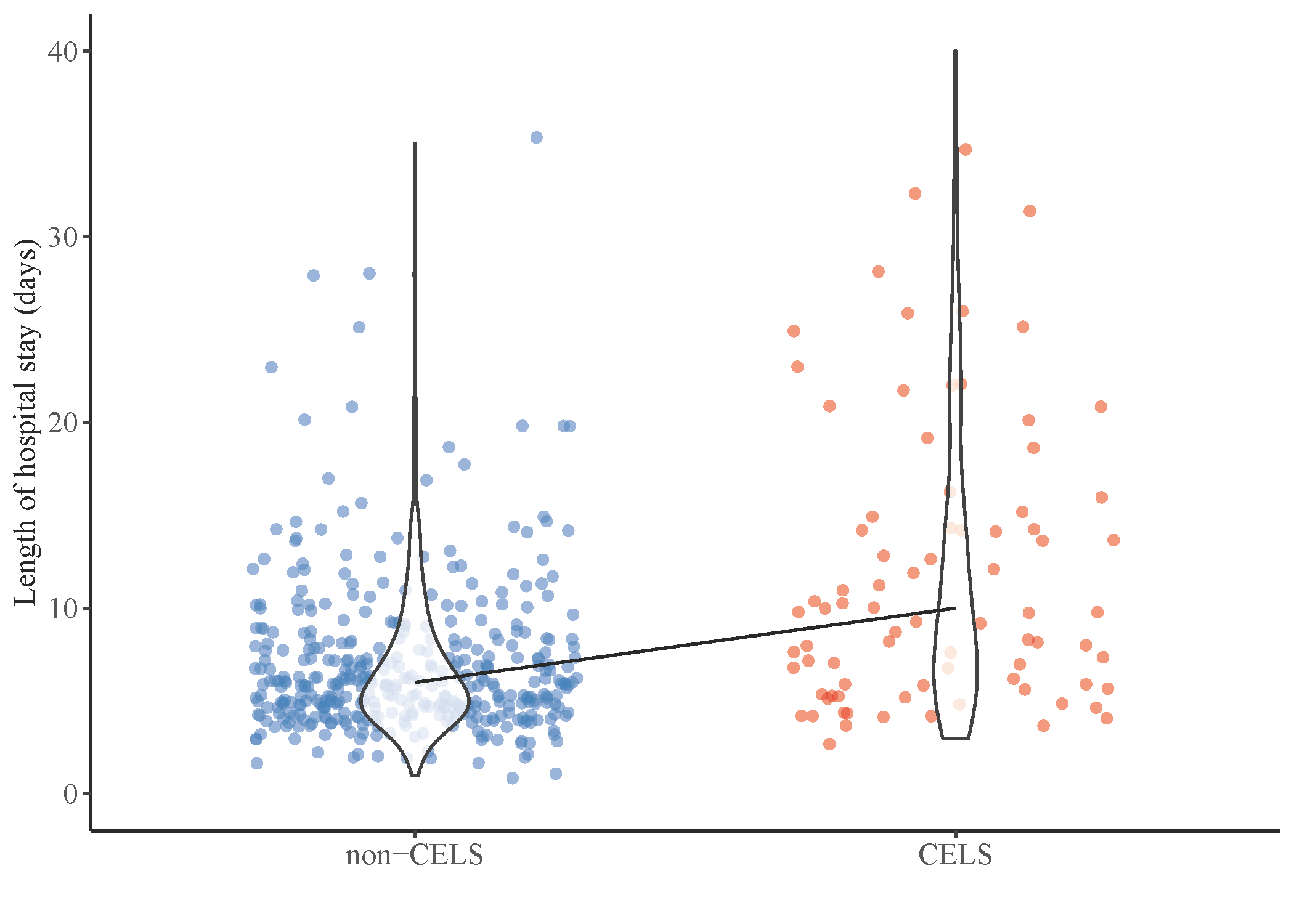

Supplement: Supplementary file 3 — Supplementary file3 (TIF 323 KB) [file 10434_2025_16965_MOESM3_ESM.tif]

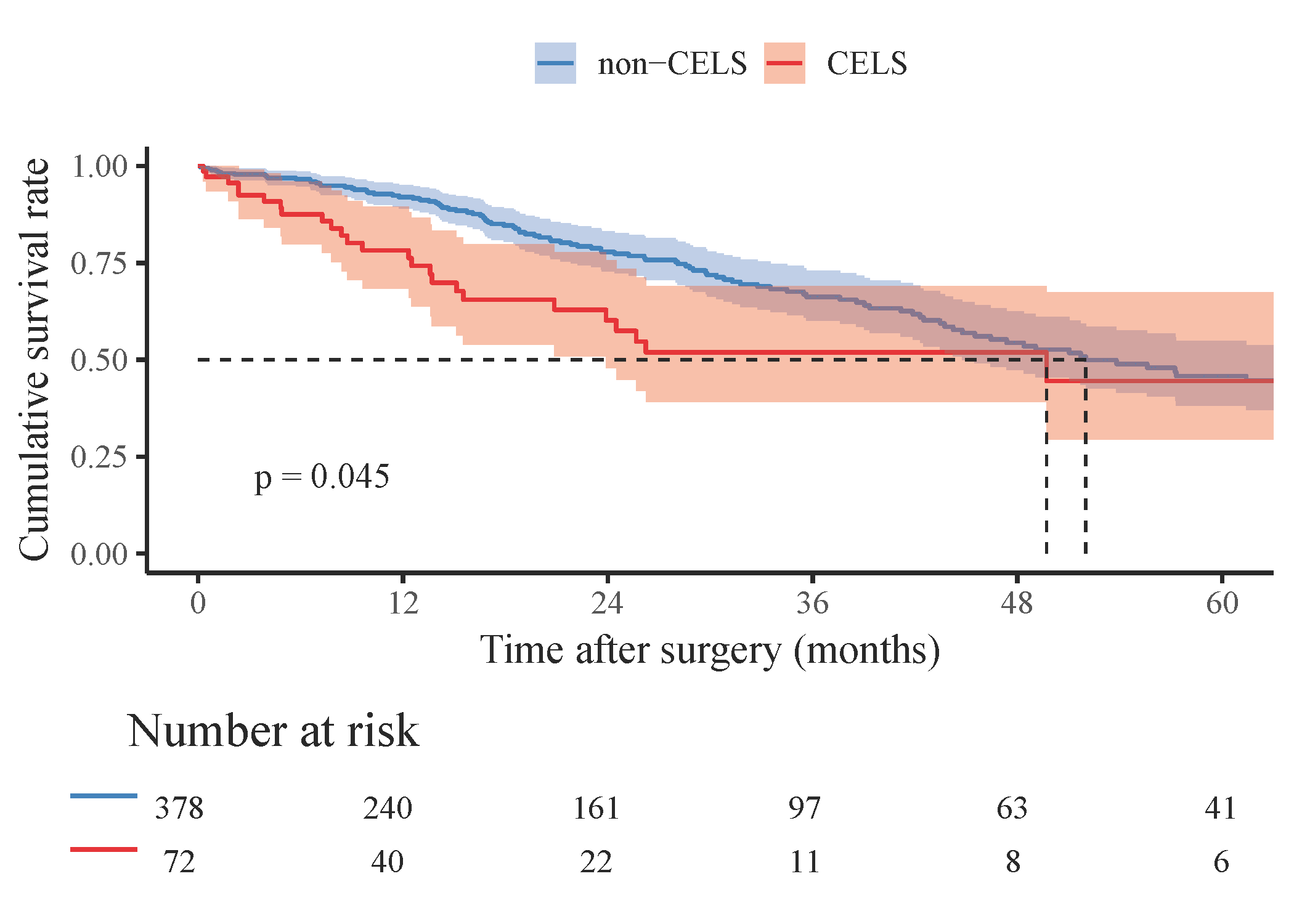

Supplement: Supplementary file 4 — Supplementary file4 (TIF 318 KB) [file 10434_2025_16965_MOESM4_ESM.tif]

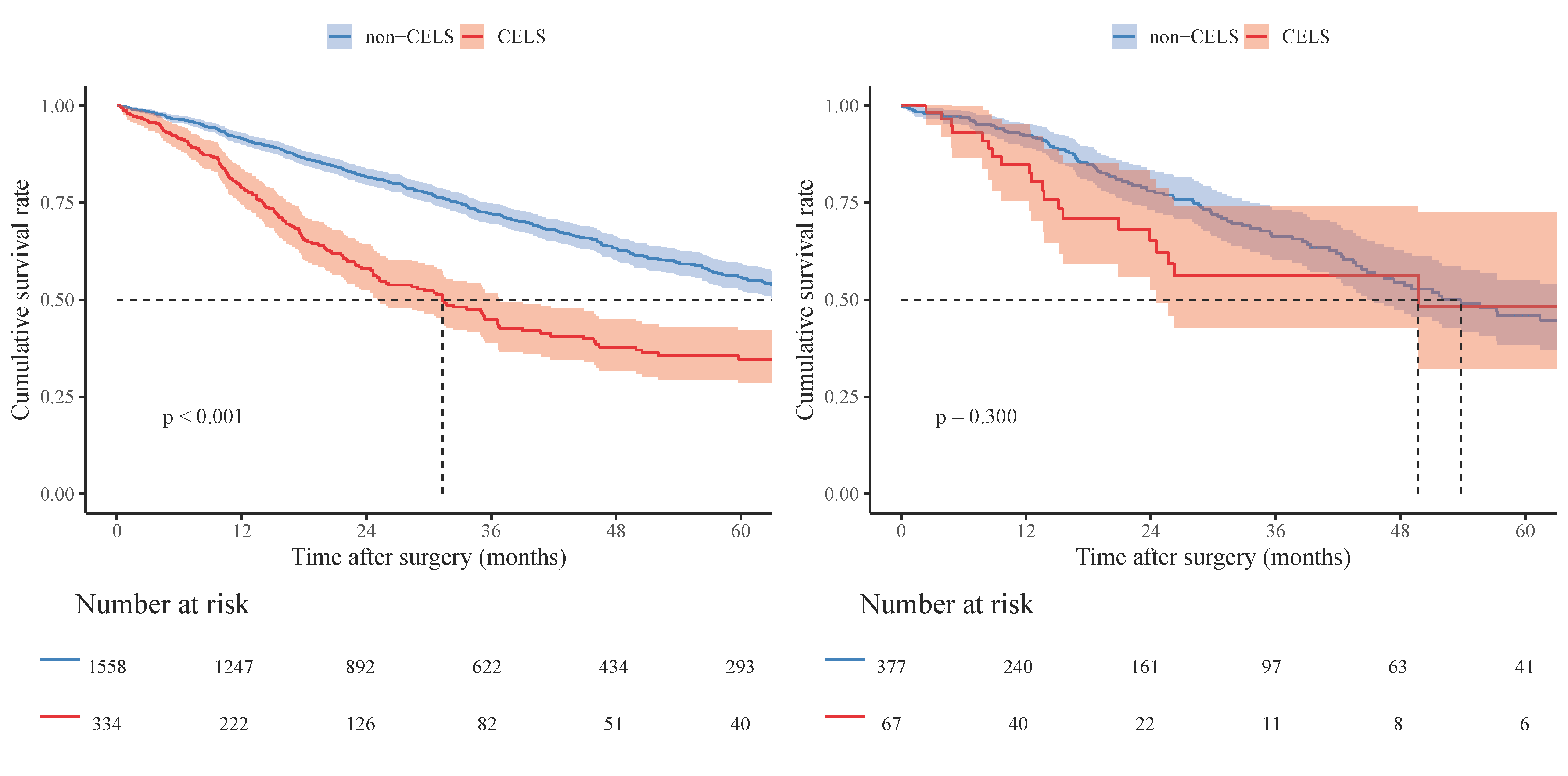

Supplement: Supplementary file 5 — Supplementary file5 (TIF 671 KB) [file 10434_2025_16965_MOESM5_ESM.tif]
